# The Triple P System of Evidence-Based Parenting Support: Past, Present, and Future Directions

**DOI:** 10.1007/s10567-023-00441-8

**Published:** 2023-07-11

**Authors:** Matthew R. Sanders

**Affiliations:** Parenting and Family Support Centre, The University of Queensland, Brisbane, QLD 4072 Australia

**Keywords:** Parenting, Population-based approach, Evidence-based parenting support, Prevention of child mental health problems; child maltreatment

## Abstract

Triple P is an integrated, multi-level system of evidence-based parenting support designed to promote the well-being of children and families to reduce prevalence rates of social, emotional, and behavioral problems in children and adolescents and to prevent child maltreatment. The system developed gradually over four decades to address the complex needs of parents and children from diverse family, socioeconomic and cultural backgrounds. It blends universal and targeted programs, a focus on developing parental self-regulation capabilities, and adopts a life span perspective with a population health framework. The Triple P system is used as a case example to discuss the past, present and future challenges, and opportunities involved in developing, evaluating, adapting, scaling and maintaining a sustainable system of evidence-based parenting intervention. Seven stages of program development are outlined from initial theory building and development of the core parenting program through to the sustained deployment of the intervention system delivered at scale. The importance of ongoing research and evaluation is highlighted so that different programs within the system evolve and adapt to address the contemporary concerns and priorities of families in diverse cultural contexts. A well-trained workforce is essential to deliver evidence-based programs, in a need-responsive manner that blends both fidelity of delivery and flexibility and is tailored to respond to the needs of individual families and local context. Programs need to be gender-sensitive, culturally informed, and attuned to the local context including relevant policies, resources, cultural factors, funding, workforce availability and their capacity to implement programs.

## Introduction

Growing international advocacy for the widespread implementation of evidence-based parenting support is based on clear evidence that positive parent–child interactions and nurturing relationships lay the foundations for a family environment that will produce healthy well-adjusted children with the life skills needed to thrive (Doyle et al., 2022; National Academies of Sciences, Engineering, and Medicine, 2016; Prinz et al., 2009; ; WHO, 2022). This advocacy for evidence-based parenting support (EBPS) places heavy emphasis on the critical role of parent–child interactions, associated parenting practices, and family relationships as potentially modifiable determinants of children’s social and emotional well-being and adjustment. These assumptions are supported by epidemiological, correlational, experimental, and randomized trials evidence showing that positively changing the parent–child relationship through EBPS programs improves child outcomes (Prinz et al., 2009; Sanders & Morawska, 2018; Sanders et al., 2014). Multiple meta-analyses and qualitative studies of parenting programs based on social learning and developmental theory confirm that modifying parenting practices based leads to significant sustained improvements in children’s behaviors and adjustment (Furlong et al., 2012; Jeong et al., 2021; Nowak & Heinrichs, 2008; Piquero et al., 2016; Sanders et al., 2023b; Solomon et al., 2017; Spencer et al., 2020), parenting skills, knowledge and confidence (Sanders et al., 2023b; Solomon et al., 2017), parental wellbeing and mental health (Furlong et al., 2021; Law et al., 2014) and reduces risk of child maltreatment (Gubbels al, 2019; Van der Put et al., 2018).

Growing evidence also shows that parenting interventions developed in high-income countries (HIC’s) can be adapted and successfully transported to low- and middle-income countries (Gardner et al., 2016; Turner et al., 2020). Although there are non-responders to all programs, positive parenting programs appear to work for many parents who participate and improve outcomes for a wide range of children including children from infancy through to adolescence.

This paper uses the development of the Triple P-Positive Parenting Program over four decades from 1978 to 2023 (Sanders, 2012) to illustrate a systematic programmatic approach to program development to identify the critical challenges, obstacles, and potential solutions associated with designing, evaluating, adapting, implementing and sustaining at scale a multi-level system of EBPS based on public health principles. This paper differs from earlier theoretical overviews of the Triple P system in this journal (e.g., Sanders, 1999) by including 1. A more detailed discussion of the historical context for the selection of the core principles and strategies of positive parenting used in Triple P and the iterative phases used to design and scale the Triple P system of intervention; 2. A discussion and appraisal of the key learnings, obstacles, and challenges related to successfully implementing the Triple P system, and 3. Discussion of challenges and future directions for the ongoing development and improvement of EBPS as a public health intervention. Throughout there is an emphasis on the relevance of our experience and learning for the wider field of EBPS and evidence-based practice more generally.

## The Past: Historical Context

As founder of the Triple P-Positive Parenting Program, the author has led a team of clinical researchers and former graduate students who collectively developed the Triple P system as a multi-level, population-based model of EBPS. The research and development base for the Triple P has been the Parenting and Family Support Center (PFSC) at the University of Queensland. The PFSC’s current strategic plan (2022–2026) includes the following goals 1) to improve the lives of families and children; 2) to create healthy, non-violent, family-friendly communities; 3) to make high-quality, culturally informed, evidence-based parenting programs accessible for all families and 4) to translate important research findings into policy and practice. These aspirations have provided a unifying values-based call to action for our research team. As a self-funded University research center, the PFSC has been fortunate to attract some outstanding academic colleagues, research staff, graduate students and eventually industry partners that have been committed to developing, evaluating and scaling EBPS programs.

### Why is a Multilevel System of Evidence-Based Parenting Support Needed?

Triple P extended the prevailing models of individual, and group behavioral parent training based on social learning principles developed in the 1970s and 1980’s. Early parenting interventions developed by pioneers such as Patterson, Hanff, which were further developed by Forgatch, Chamberlain, Webster-Stratton, Forehand & McMahon, and Eyberg while effective, reached relatively few families. To reduce the prevalence rates of child social, emotional, and behavioral problems and child maltreatment a more comprehensive, integrated, multilevel system of parenting support from birth through to adolescence based on population health principles was required (Sanders, 1999). However, in the 1980’s there was little consensus or experience to guide what might be involved in developing, evaluating, implementing and sustaining a comprehensive public health approach to parenting support as the emphasis in both research and in policy had been on adopting a targeted approach by focusing on the most vulnerable, “at risk” children or families. Most evidence-based prevention models such as “Incredible Years”, “Fast Track”, “Nurse-Family Partnership”, and “Parent–Child Interaction Therapy” focused on targeting high-risk or vulnerable children.

The notion of a having a suite of evidence-based interventions on tiered continuum of increasing strength or intensity and narrowing population reach had little policy or empirical support in the field of parenting. The principle of “minimal sufficiency” that emphasized a parent should be offered as much or as little support with parenting as they may require to resolve a difficulty was new. Indeed, the notion of using brief, low intensity interventions was criticized in some quarters as endorsing “inexpensive” but ultimately insufficient intervention options to address the complex needs of vulnerable and “hard to reach” parents.

From the outset it was clear that a “one-size fits all” approach (e.g., a 10–20 session group program) would not work or be accepted by the field. Hence, for many years we worked concurrently on developing and testing both universal and targeted interventions. We worked on programs with wide population reach such as television series on parenting such as the “Families” TV Series in New Zealand on TV 3 (e.g. Sanders et al., 2000) and in the United Kingdom on “Driving Mum and Dad Mad” on ITV (Sanders et al., 2008), podcasts on parenting on ABC in Australia (Morawska et al., 2014) and during the COVID-19 Pandemic podcasts and TV series on “Parenting in a Pandemic” (Morawska et al., 2022). From the mid 1980’s to the present we developed a range of variants targeting specific at-risk or vulnerable groups (e.g., parents of children with developmental disabilities, divorced or separated parents, parents with marital discord, single parents, parents with mental health or problems of addiction, and indigenous parents). We recognized early that different intensities of intervention support would be needed across a community and for the same family at different stages of their own individual parenting journey. For example, a parent with a four-year-old with severe early onset conduct problems might initially require an intensive multi-session individual or group program. If this early intervention was successful, the same parent might benefit from a lighter touch approach when the target child reaches elementary (primary) school or high school or for other siblings.

**Fig. 1:**
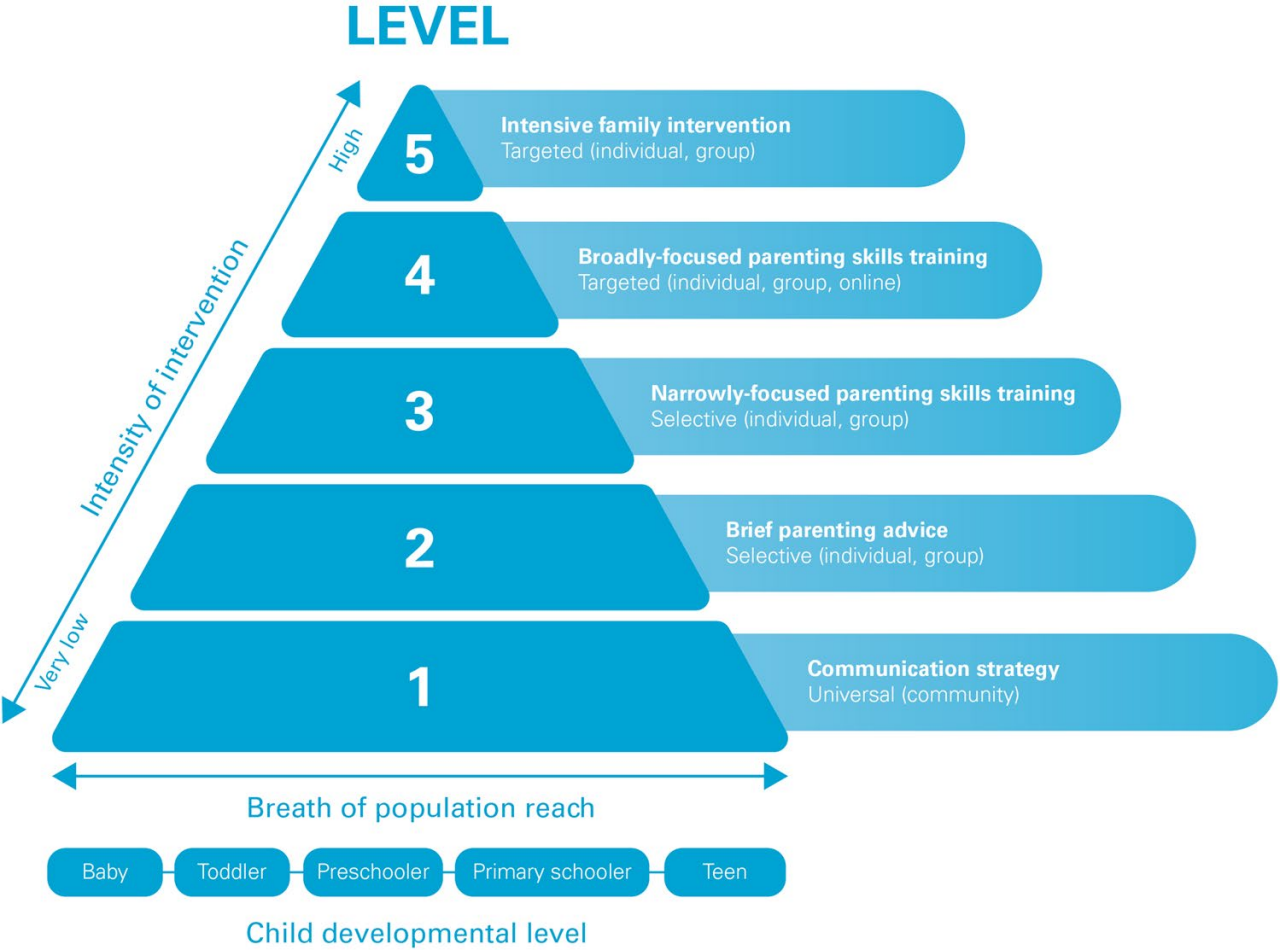
The Triple P Multi-level System of Parenting Support

Figure 1 depicts the essential features of the Triple P multilevel system. In a community with a fully developed suite of Triple P programs, all five levels of intensity of the intervention system would be available to parents so they could engage, complete and then re-engage in a program that meets their need and capacity to participate. As the intensity of program options increases from level 1 to level 5 the costs per participating parent understandably increases due to additional staff time involved in delivering the intervention, increased training costs for more intensive programs and because more severe or complex cases require more intensive levels of support, and the level of program reach reduces. This multilevel approach is not a traditional “stepped care” model where parents participate in lower intensity programs prior to receiving more intensive programs. From the outset parents should receive the level of support they require to solve a problem and have the capacity to engage in. A parent’s involvement with Triple P may begin with a more intensive program (levels 4 or 5) and then decrease over time for any subsequent involvement (levels 1–3) if required. The system is designed to avoid inadvertently creating dependency or reliance on Triple P for ongoing parenting support. It aims to provide the minimally sufficient support that enables the parent to independently “get on with it” and raise their children without ongoing reliance on external professional or peer support. The system includes EBPS programs offered from infancy through each successive stage of development to adolescence, the system is designed to enable a parent of children at any age to participate. For each age group, there is a mix of lower and higher intensity programs, individual, group or online programs so that the level of support offered can be appropriate to need.

Our approach to developing new variants of Triple P for different populations is based on whether an existing program (e.g., Standard or Group Triple P) could be successfully applied to a different or new population or problem without substantial modifications. However, clinician and parent feedback sometimes indicated that certain populations required different or additional program components or supports to address additional risk or protective factors (e.g., parents of children with ASD, parents at risk of child maltreatment, parents with relationship conflict). This end-user viewpoint typically provided the impetus for developing new program variants such as Stepping Stones Triple P for parents of children with a disability, Pathways Triple P for parents at risk of harming their children, Resilience Triple P for children who were bullied and Fear Less Triple P for parents of children with anxiety disorders. There are currently 33 discrete empirically supported Triple P programs that comprise the Triple P System (see Table 1 and Sanders & Mazzucchelli, 2017).

**Table 1:** Triple P Program Variants

Program name	Program name	Families targeted
Communications Campaign (level 1)	Stay Positive	All families
Universal Seminar Programs (level 2)	Seminars 0–12 Triple P, Teen Seminars Triple P, Stepping Stones Triple P Seminars, Fear-Less Triple P Seminars, Lifestyle Triple P Seminars, Disaster Recovery Triple P Seminars	All parents of typically children from birth to adolescence and parents of children with developmental disabilities
Primary Care Programs (level2)	Primary Care Triple P, Teen Primary Care Triple P, Stepping Stones Primary Care Triple P	All parents of typically developing children and children with developmental disabilities
Discussion Group Programs (level 3):	Discussion Groups Triple P, Teen Discussion Groups Triple P	Parents of preschool, elementary school and teenage children
Standard Programs (level 4):	Standard Triple P, Standard Teen Triple P, Standard Stepping Stones Triple P	Parents of children with moderate to severe social, emotional and behavior problems
Group Programs (level 4)	Group Triple P, Teen Group Triple P, Stepping Stones Group Triple P, Baby Group Triple P, Fear Less Group Triple P, Life Style Group Triple P, Resilience Group Triple P	Parents of children with conduct problems, developmental disabilities, infants, anxiety problems, overweight and obesity, who have been bullied (peer victimized)
Enhanced Programs (level 5):	Enhanced Triple P, Pathways Triple P, Family Transitions Triple P, Family Life Skills Triple P	Parents with mental health problems, relationship difficulties with partners, high levels of stress, at risk of abusing or neglecting children, experiencing separation or divorce, parents, histories of trauma or adverse childhood experiences (ACES)
Online programs (level 2–4):	Triple P Online, Baby Triple P Online, Fear-Less Triple P Online, Teen Triple P Online, Stepping Stones Triple P Online, Family Transition Triple P Online, Resilience Triple P Online, Play Well Triple P Online	Online versions of selected group programs (above) plus Play Well Triple P a universal to promote positive sports parenting

Global events such as COVID-19 pandemic and concerns about natural disasters because of climate change provided the impetus to develop new program resources. For example, during the early stages of the pandemic in 2020 we developed a range of free online resources (https://pfsc.psychology.uq.edu.au/covid19-resources) relating to parenting in a pandemic including downloadable tipsheets, parent guides, a 20-episode podcast series (Turner & Sanders, 2020) and a 12-episode television series “Parenting in a Pandemic” (Morawska et al., 2022). When the Triple P system of EBPS is fully implemented it consists of a communication/ information campaigns to promote positive parenting such as “Stay Positive” (Wilkinson, 2017), age, topic, and problem specific tip sheets, parent workbooks and video resources for seminar programs, primary care consultations, discussion groups and more intensive multi-session individual or group programs, or as delivered through self-guided online parenting programs (e.g. Triple P Online, Fear Less Triple P Online, Teen Triple P Online). Having continuous support in using differing levels of intensity of interventions appropriate to the parent’s capacity and needs ensures that relevant, developmentally appropriate, and helpful information and support can be provided when and where it is needed. When flexible delivery options are available (e.g., individual, group, telehealth, self-guided online programs) parents are more likely to engage.

### Adopting a Systems-Contextual Approach

Triple P adopts a broad systems-contextual or ecological perspective in supporting parents raise healthy, well-adjusted children (Bronfenbrenner, 1986; Sanders & Turner, 2018). A systems-contextual approach (Sanders et al., 2017) ensures that the multiple determinants of parenting capability are factored into the design of the parenting support systems and recognizes that broader contextual and cultural factors such as chronic and intergenerational poverty, social disadvantage, high mortality, marital instability, social isolation, crime and alcohol and other drug addictions can influence parental capacity (Bradley & Corwyn, 2005; Sanders & Turner, 2018). The approach also factors in workforce issues such as the training and supervision of providers, organizational leadership, policy advocacy, and funding.

From the core program several new program variants have been developed and included in the Triple P system if there was sufficient empirical evidence to justify its inclusion. Adherence to this principle has undoubtedly slowed down the release of programs which had to wait until sufficient evidence accumulated to support its release. On occasions we decided not to release programs after a trial because the evidence was insufficient to include in the model. Taking Care Triple P, the variant developed for foster carers was an example of this (Job et al., 2022). However, conducting adequately powered clinical trials is a slow and expensive process (from initial grant writing to completion and publication of the trial and with limited Government funding for parenting and family intervention research through major funding agencies (see Havighurst et al., 2022), we now release promising programs to encourage independent evaluations. The decision to release a new program for inclusion in the Triple P License agreement is made by the program authors. However, the decision to disseminate the program is made by UQ’s industry partner (TPI) in consultation with the PFSC. Program release considers the wider commercial realities such as having an identified workforce to train and implement the program and the likely demand for training.

The Triple P system is often deployed as part of a place-based intervention targeting vulnerable geographical areas involving multiple disciplines across health, mental health, education, the social care/child welfare and community sectors. This multi-sector involvement enhances the capacity for consistency of messaging about parenting in a community and to increases the potential reach of the intervention as it is being delivered across different service delivery contexts. Developing a sustainable, place-based system of evidence-based parenting support involves a complex, iterative process that involves synergistically bringing together the key drivers of systems-level change. It involves developing respectful, collaborative partnerships between program developers, purveyor organizations, university/research organizations, governments, industry partners, service providers, and the wider community. In indigenous communities and First Nations communities, it can involve extensive community consultation with elders, indigenous practitioners and parents as consumers (see Turner et al., 2018, 2020) for a discussion of cultural adaptation processes used to introduce Triple P into First Nations and Indigenous communities in Australia and New Zealand.

### Iterative Developmental Process (1978–2023)

This section documents the seven overlapping phases of research and development activity involved in designing the Triple P system. Table 2 summarises the main activities and outputs associated with each phase. The seven-phase model discussed below evolved through an iterative process that involved a mix of a retrospective task analysis of what was done, with a prospective process that involved pre-planned forecasting of what needed to be done based on evolving evidence from implementation science and learning derived from practical experience.

**Table 2:** Iterative Developmental Process of Triple P

Parenting program content and intervention procedures	Professional training system	Outcome assessment tools	Scaling and dissemination strategy	Implementation framework	Quality improvement framework	Knowledge transfer strategies
Developing a Theory of change and core principles of positive parenting	Developing of a theory of change for professional training	Developing program specific assessment tools	Identifying an industry partner for dissemination	Developing an implementation framework	Developing procedures to manage Conflicts of Interest (COI)	Hosting monthly PFSC Research Seminars
Parent workbooks, tipsheets, flipcharts, videos, and online programs	Developing course content for multi-disciplinary practitioner training course	Developing of Automatic Scoring and Reporting System (ASRA) and fidelity monitoring tools	Establishing a licensing agreement between UQ and TPI	Establishing a Program Implementation and Evaluation Support (PIES)	Establishing of Triple P Provider Network (TPPN)	Organizing biennial Helping Families Change Conference (HFCC)
Practitioner kits (practitioner manuals)	Participant notes, slide presentations, videos	Designing an International Parenting Survey (IPS)	Marketing and program promotion	Developing procedures for cultural adaption	Establishing a Triple P Trainers Network (TPTN)	Contributing to Regional Triple P Updates, Triple P Master Classes
Participant notes, PowerPoint presentations, video materials	Triple P Training Curriculum Development Committee, trainer course	Developing open access assessment tools repository (ATR)	Establishing a global Triple P dissemination network	Developing a supervision model (PASS-Peer Assisted Supervision and Support)	Conducting surveys to gauge views of parents and practitioners	Supporting International Congress on Evidence-based Parenting Support

### Phase One: Development of the Core Positive Parenting Program Curriculum (1978–1993)

The beginnings of the parenting program known as Triple P (since 1993) date back to 1978 and the foundational work undertaken by the author as part of his doctoral dissertation in psychology at the University of Queensland in 1978 (completed 1981) under the supervision of Professor Ted Glynn (the University of Auckland), Professor Jack James (the University of Queensland) and advisor Professor Todd Risley (University of Kansas). Triple P began as an individually administered 10-session home coaching model of parent training for parents of preschool-aged children with disruptive behavior problems (Sanders & Glynn, 1981). The foundational work focused on teaching parents to use positive parenting and contingency management skills to reduce disruptive behavior and self-management skills to enable parents to transfer or generalize their skills to managing children in different out-of-home settings. The focus on promoting parental self-regulation skills remains a central focus in all Triple P programs and informs the overall design of the Triple P system professional training system.

#### Theoretical Basis

The theoretical origins of Triple P were based on principles of applied behavior analysis (Baer et al., 1968), self-regulation theory (Bandura, 1986), and developmental theory particularly theories of social competence (Hart & Risley, 1995). Since that time the theoretical perspectives defining the model have evolved to incorporate other perspectives. Self-regulation theory (Karoly, 1993; Sanders et al., 2019) became a central aspect of the model not just focused on how to consult with parents collaboratively (Sanders & Burke, 2014; Sanders & Lawton, 1993), but also how to train practitioners to work with parents (Mazzuchelli & Ralph, 2019), how to train trainers who train practitioners (Ralph & Dittman, 2018), how to provide implementation support via implementation consultants to agencies (McWilliam & Brown, 2017) and the approach to policy advocacy work with Governments (McWilliam et al., 2016). Self-regulation theory (Sanders et al., 2019) provided an integrating conceptual framework that then enabled procedures used to train and coach practitioners, to supervise trained practitioners to promote fidelity of implementation (Sanders et al., 2020a, 2020b), to consulting methods used with agencies to help them clarify expectations, set goals and evaluate outcomes. The aim was to promote personal agency and independent problem solving so all actors- children, parents, practitioners, trainers, and organizations using Triple P had the knowledge, skills and personal agency to become self-determining regarding their use of Triple P as a system and overtime less reliant of external support from practitioners or in the case of agencies from TPI as a purveyor organization. This approach worked well as part of a population or community-wide public health approach to deliver parenting support where resources were limited, and interventions needed to be cost-effective.

In the late 1970’s little information was available in the parent training literature on how to actively promote the transfer of learning or generalization of parenting skills from one child to their siblings, different behaviors, settings and over time (Wells et al., 1980). My interest in “programming for generalization” effects led me to develop and test a set of procedure to teach parents self-management skills (goal setting, self-monitoring, self-evaluation of strengths and limitations) to assist parents learn to generalize or transfer their knowledge and skills to tackle different problems, in varied settings (e.g., out of home, shopping, visiting), with other siblings in the family and over time (Sanders & Glynn, 1981). The core principles of generalization programming such as “training loosely” and “training sufficient exemplars” as articulated by Stokes & Baer, 1977) were integrated into the methods used to train parents to use self-management skills. Hence, the Triple P system synergistically combined several complementary theoretical perspectives that vary in emphasis depending on the target population (e.g., parents, practitioners, agency leads, implementing organizations). No single theoretical orientation incorporated the full range of concepts and procedures needed to implement a population-based system of parenting interventions.

#### Development of Core Content

The core parenting skills used in Triple P were linked to five key principles of positive parenting: 1) Having a safe and engaging environment, 2) creating a positive learning environment, 3) using assertive discipline, 4) having realistic expectations, and 5) taking care of yourself as a parent. The specific parenting skills had four primary targets—Skills that promote a positive relationship with the child (quality time, talking with children, and showing affection), skills that encourage desirable behavior (descriptive praise, proving attention, selecting age appropriate and engaging activities), skills that teach children new skills and behavior (setting a good example, incidental teaching, and Ask, Say, Do) and skills to manage problem behavior (ground rules, directed discussion, planned ignoring, clear, calm instructions, logical consequences, quiet time and time out). In addition, to these child-focused skills, parents were taught self-management skills such as goal setting, self-monitoring and self-evaluation of strengths and challenges, setting personal goals and a parenting plan for change, planning and preparing for high risk or challenging parenting situations.

From this foundational base additional age-appropriate parenting skills were introduced into different program variants such as Teen Triple P (behavior contracting, emotional behavior routine, managing high risk situations), Stepping Stones Triple P (teaching backwards, brief interruption, diversion onto a new activity), and Triple P for baby (calming, settling techniques for crying and sleep). As Triple P evolved additional parenting skills were introduced for specific groups of children or parents. For example, Pathways Triple P for parent at risk of child maltreatment (e.g., stress management skills, attributional retraining, anger management), Enhanced Triple P (partner support and couple communication, coping with emotions), Family Transitions Triple P (e.g., conflict management skills), Lifestyle Triple P (e.g. healthy eating and physical activity). Each new iteration of Triple P incorporated procedures and strategies based primarily on contemporary cognitive behavior therapy and empirical literature supporting the use of techniques relevant to addressing the area of concern. For example, to address stress and anxiety problems techniques of cognitive restructuring, stress management such as relaxation, mindfulness, deep breathing was employed and integrated where possible into everyday parenting tasks, routines and situations (e.g., early morning rush of getting ready for school or work).

#### Contributors

Thirteen individual co-authors contributed to the development of Triple P program materials. These program developers have mostly been the author and former doctoral students (now colleagues). Most were studying clinical psychology at the University of Queensland and a small number of non-student academic colleagues were affiliated with the PFSC. The program developers have included Karen Turner, Carol Markie Dadds, Alan Ralph, Alina Morawska, Felicity West, Vanessa Cobham, James Kirby, Helen Stallman, Trevor Mazzucchelli, Lisa Studman, Carmon Spry, Karyn Healy, and Aileen Pidgeon. The program was gradually extended beyond the concerns of parents of preschool-aged children to other age-groups of infants, toddlers, elementary (primary) school-age children and teenagers and different types of child problems (e.g., parents of children with a disability and chronic health concerns) and family contexts (parenting in the context of separation and divorce, parents with problems of addiction or mental health, grandparents).

A range of implementation tools and resources were developed for different variants to support all aspects of implementation, scaling, and sustainment, including parent materials, written and video resources used in workforce training, supervision procedures to support practitioners implementing Triple P, self-report and observational measurement tools for tracking client outcomes, fidelity assessment tools for promoting and monitoring fidelity, practitioner and trainer networks to support staff involved in dissemination, and quality assurance methods to review program developments and revisions, science communication strategies. A commercially viable system of training and dissemination was developed and then refined with TPI as industry partners so the program could be scaled internationally.

#### A Lifespan Perspective to Parenting Support

A lifespan perspective enables the parenting competencies associated with each successive phase of a child’s life to be identified and targeted. Some parenting skills (e.g., praise and positive attention) are continuous and apply to each subsequent phases of development. Others are more discontinuous and are particularly relevant at certain times in specific phases (e.g., quiet time or timeout for preschool-age children). A lifespan perspective recognizes that parents have a continuing but changing role in the lives of their children as they grow into adults and transition to parenthood themselves and producing grandchildren and eventually great grandchildren. For many family’s grandparents have a continuing role in the promoting the wellbeing of children. Both non-custodial and custodial grandparents play a vital role in caring for, educating and supervising grandchildren. Several trials have shown that Triple P is effective with both non-custodial grandparents providing occasional care (Kirby & Sanders, 2014) and for custodial grand families who are the child’s guardian and provide fulltime care (Smith et al., 2018).

#### Levels of Intervention

Research into the effects of parenting programs shows that both low-intensity and higher-intensity parenting programs can work for particular problems. Having different levels of parenting support available provides options for varying the intensity of support (amount of contact, number of sessions, amount of material covered) parents receive to solve a problem. Support can range from providing information and minimal skills training and coaching to providing more intensive practice of skills and/or providing additional range of skills training or coaching when working with parents with multiple additional problems (e.g., substance abuse, mental health problems, relationship difficulties). For example, both Primary Care Triple P (level 2) and Standard Triple P (level 4) introduce parents to strategies for encouraging desirable behavior and for preventing and managing problem behavior. In Primary Care Triple P this is done with the use of tip sheets in 2–5 sessions (2–5). In Standard Triple P more sessions (10 sessions) are involved where the same skills are practiced but applied to a wider range of child problem behaviors. At the highest level of intensity, Enhanced Triple P (level 5) introduces parents across ten sessions to additional cognitive-behavioral skills such as stress and mood management, partner support, effective couple communication and conflict management skills, anger management, and attributional retraining. In Family Life Skills Triple P sessions also covers dealing with emotions, relationships, self-care, past trauma, healthy lifestyles and planning for the future (Sanders et al., 2023a).

#### Flexible Delivery

Traditional parenting training programs are delivered through multi-session programs either in groups or in individual sessions. These delivery options have been extended in Triple P to include telephone and telehealth methods of delivery using zoom and other video technologies and through multiple interactive self-guided online programs. An important study by Prinz et al (2022) used a randomized non-inferiority design to compare “in person” individually delivered Standard Triple P and Triple P Online as a self-guided program with no professional help in a sample of children with early onset conduct problems. Results showed the online program and Standard Triple P delivered with high fidelity by clinicians, achieved comparable positive outcomes were achieved at 12 months follow up in child and parent behavior. Importantly the online program was considerably less expensive to deliver than the staff implemented program (Ingels et al., 2022).

### Phase Two: Development of a Professional Training System

The predominant activity in this phase was to develop a multidisciplinary system of professional training and workforce development. The resulting model of professional training was a blend of principles and strategies to promote practitioner self-regulation (reflective practice) combined with active skills training methods that included 1–5-day training courses that incorporated video and live modelling of practitioner skills, small group practice, feedback and coaching and competency-based assessment and a knowledge quiz. This system of training involved the development of a range of high-quality resources for use by trainers in running training courses (including videotaped demonstrations of core consultation skills, practitioner participant noters, practitioner manuals and parent resources). Evaluation studies of the effects of Triple P training have shown high satisfaction with training and improved self-efficacy regardless of level or variant of training, country where it is conducted, type of discipline involved (Sanders et al., 2023a; Sethi et al., 2014). Several studies have shown that the training courses receive Following initial training practitioners are encouraged to participate in Peer Assisted Supervision and Support (PASS) sessions to promote program fidelity and use (McPherson et al., 2016; Sanders & Murphy-Brennan, 2013). PASS sessions use a mix of reflective practice, use of self-regulation skills and peer assisted learning principles that provide practitioners with constructive individualized case-based feedback from peers. A PASS session video demonstration and a workbook have been developed to provide guidance to practitioners on how to incorporate PASS into routine clinical practice (Sanders & Murphy-Brennan, 2013). A systematic evaluation of PASS (McPherson et al., 2016) showed that participation in PASS sessions increased practitioner self-efficacy in delivering Triple P to parents.

### Phase Three: Development of Clinical Assessment and Monitoring Tools

Even though an intervention may be supported by clinical trial evidence showing the program works with families in the study sample, it does not mean that the program achieves the same outcomes when used with clients the practitioner is working with. Successful implementation of an evidence-based program requires practitioners to routinely use tools that can reliably assess change over time in relevant client outcomes The outcomes produced can be the same or similar, better, or worse than the original studies particularly if different measures are used. Consequently, each program variant of Triple P includes outcome assessment tools that can be used by practitioners to collect systematic data on their own client outcomes. In this way practitioners are encouraged to evaluate all Triple P programs they run. Over time substantial practice-based evidence can evolve so that agencies can determine for themselves whether the program is achieved desired outcomes. However, agencies need to dedicate resources and personnel to analyse and report on outcomes achieved. Examples of reliable and change sensitive tools developed to support the evaluation of Triple P include the CAPES (Child and Parent Efficacy Scale; Morawska et al., 2014) and the PAFAS (Parent and Family Adjustment Scale; Sanders et al., 2014) and Parental Self-Regulation Scale, (PSRC, Tellegen et al., 2022). All PFSC developed assessment tools are open source and can be downloaded from our Measure Library website (https://pfsc.psychology.uq.edu.au/research/measures-library).

### Phase Four: Development of a Dissemination and Scaling Model

Once the model of intervention and a method of training practitioners was developed, a theoretical model of dissemination for scaling Triple P was developed between 1994 and 1998. At the time all dissemination of Triple P was handled through the Parenting and Family Support Centre and there was no road map or similar examples of behavioral science innovation that had been successfully disseminated on a global scale. Although programs such as Incredible Years were developed around the same time, Triple P developed its own approach to dissemination. It quickly became apparent that the PFSC did not have the capacity or business model to deliver professional training at scale in a sustainable way. The PFSC was primarily a clinical research and training facility. The model eventually adopted required program authors to assign their intellectual property rights to the University of Queensland (their employer) so the university’s main technology transfer company UniQuest Pty Ltd could license a dissemination or purveyor organization to disseminate the program globally on behalf of the PFSC authors and University. Triple P International Pty Ltd entered an exclusive license agreement with UQ to further develop and scale Triple P through a commercially viable training and dissemination model. The license agreement required TPI to pay a royalty back to the University which was split equally according to University intellectual property policies between University of Queensland (UniQuest), Faculty of Health and Behavioural Sciences, the School of Psychology’s Parenting and Family Support Centre and contributory authors. Having an industry partner being responsible for dissemination of Triple P ensured that a continuous revenue stream from dissemination (sales of training, implementation support and resources) was available to support the ongoing research and development mission of the PFSC. TPI developed as “one stop shop” organization responsible for all aspects of dissemination including publishing of written materials, video production and development and maintenance of online platforms, contracting and delivery of professional training and implementation support. Having a single industry partner dedicated to management of the global dissemination of Triple P was crucial to scaling the program in an economically sustainable manner and for ensuring there was ongoing investment in the program. Most publishing operations do not have an integrated suite of services needed to scale a program.

**Fig. 2:**
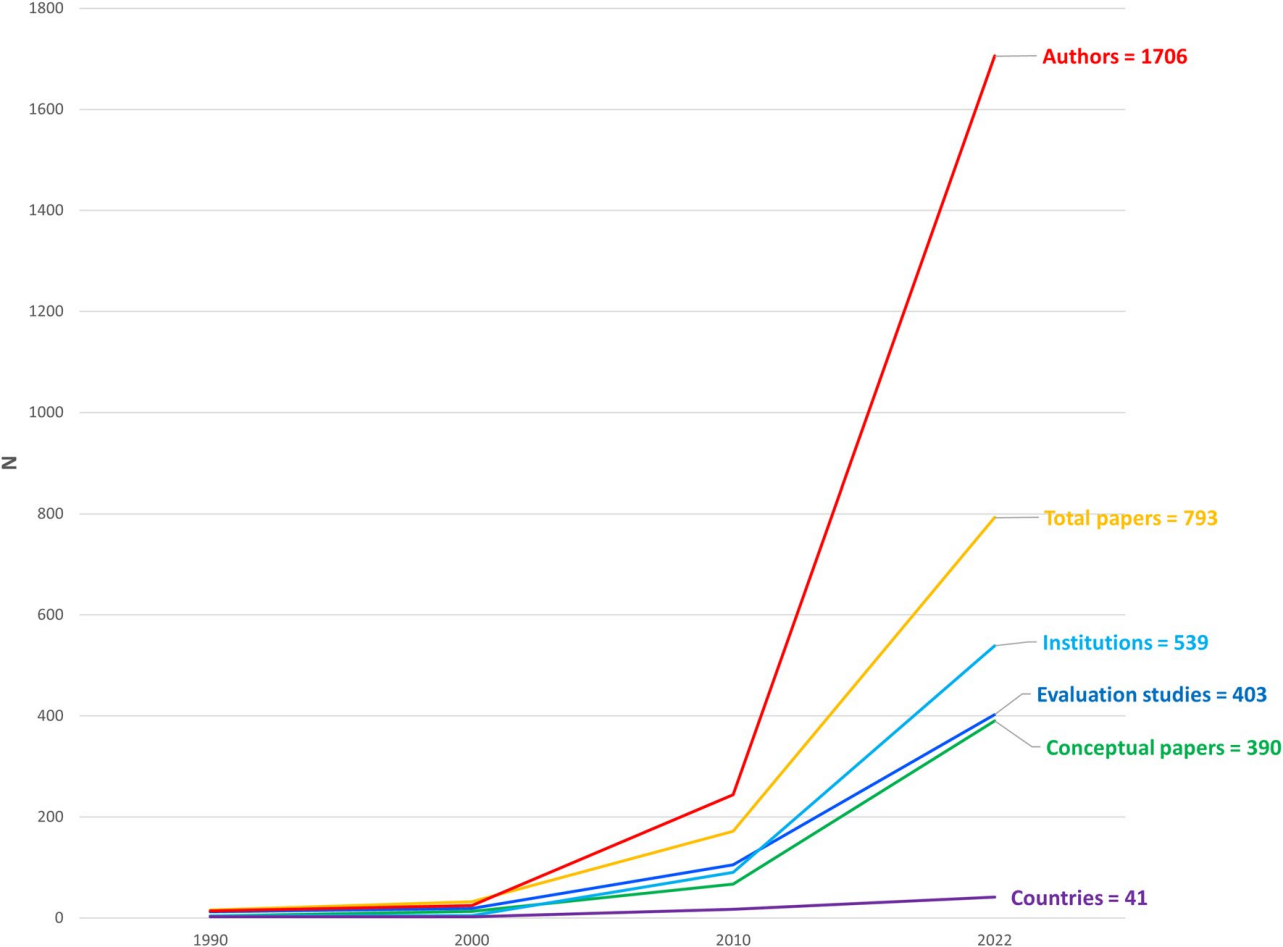
Growth of Triple P Research

Overall, the dissemination and scaling model has been very successful with over 100,000 unique practitioners having been trained in Triple P, from 72 countries around the world. An important by-product of having a dedicated dissemination organization is that it made the program and associated training is accessible to other research groups. Figure 2 plots the growth of Triple P indices of academic outputs over a period of four decades. A clear acceleration in growth was associated with the commencement of the TPI license agreement in 2001. This enhanced accessibility contributed to the growth of scholarship relating to Triple P including peer reviewed articles, independent evaluations of Triple P including RCT’s, the development of international collaborative research partnerships and the promotion of research training opportunities for higher degree students interested in parenting and family intervention. Between 1996 and 2021 58 doctoral theses devoted to Triple P were conducted by PFSC higher-degree students. Most evaluation studies evaluated Group Triple P (a level 4 intervention), although studies were conducted on all disseminated programs across all five levels of interventions. This cumulative international research activity has resulted in 793 papers, involving over 1706 named investigators, from 539 academic institutions across 41 countries. The establishment of a dedicated dissemination mechanism in 2021 through Triple P International greatly accelerated the growth of research outputs including independent evaluations of Triple P. The growth of Triple P research outputs across four decades is plotted in Fig. 2.

### Phase Five: Development of Implementation Framework

Having a theoretically informed, high-quality, evidence-based system of intervention and a system of professional training is no guarantee that the program will be implemented by trained practitioners with fidelity over a sustained period. The Triple P Implementation Framework (McWilliam et al., 2016) was developed to guide TPI as a purveyor organization and its implementation consultants who provide post-training support to organizations who had staff trained to implement Triple P. This implementation framework applies two core Triple P principles-minimal sufficiency and self-regulation as drivers of change. The model involves a five-stage process including 1) engagement, commitment and contracting, 2) implementation planning, 3) training and accreditation, 4) implementation, and 5) maintenance. The framework aims to provide the sufficient level of implementation support necessary to enable the organization to independently self-manage their implementation of the program in a sustainable manner.

### Phase Six: Development of a Quality Improvement Framework

Quality improvement strategies have been embedded into both the PFSC’s and TPI’s operational procedures. As program developers/clinical researchers, the PFSC has had to develop Conflict of Interest (COI) Management policies and procedures consistent with the University’s research quality framework, requirements of funding bodies, and calls for greater transparency in the field generally (Sanders, 2015; Sanders et al., 2019). This has included routine declaration of conflicts of interest in publications and presentations involving Triple P authors, adopting procedures to minimize bias in reporting outcomes including clinical trial registration, reporting of any null findings and where funds are available use of independent data analysts. The PFSC has also conducted internal audits reviews of the Centre’s operations, developed a strategic plan for continuous improvement and conducts routine evaluations of all training conducted by center staff. Focus groups and online parent surveys such as the online “My Say” Surveys of parents of children with a disability (Einfeld et al., 2018). TPI prepares regular quality assurance reports for the University of Queensland (via UniQuest) that report on training outcomes of all Triple P training courses using standardized training evaluation measures. A Triple P Provider Network (TPPN) supports practitioners trained to deliver Triple P with updates and downloadable resources needed to deliver the program to parents. All Triple P Trainers have continuing education requirements and belong to the TPI Trainers Network (TPTN) which schedules regular trainer updates on new developments and research findings, and PASS supervision sessions. There is also a Curriculum Development and Review Committee (CDRC) that reviews all new course materials for professional training, a Program Practice Advisory Panel (PPAP) that reviews all new program materials and provides feedback to authors before a program is published and disseminated. The PPAP is comprised of mainly of trainers and implementation consultants but are independent of the PFSC developers. These quality assurance mechanisms are designed to reduce the risk that program materials and resources being released that are not dissemination ready.

### Phase Seven: Development of Knowledge Transfer Strategies

Effective knowledge transfer is essential for EBPS programs to impact on policy and practice. We have made extensive efforts to share the learnings and findings arising from research and our experience in the dissemination and implementation of Triple P. A dedicated searchable webpage provides regular updates of Triple P research that have been published (www.pfsc.uq.edu.ua/evidence). A notable feature of this knowledge transfer procedure is the involvement of a diverse range of researchers and research institutions around the world who have conducted research on Triple P. A 46-chapter edited volume on the Power of Positive Parenting (Sanders & Mazzuchelli, 2017) drew together a wide range of contributing authors involved in Triple P program development and/or research to comprehensively document how Triple P was designed, evaluated, adapted, and disseminated to ensure our learnings and insights are shared with other evidence-based programs.

A series of knowledge transfer strategies have been developed (sometimes in partnership with other organizations) to share our learnings with other practitioners, researchers and members of the public interested in the field of parenting. These have included a monthly seminar series for PFSC staff and students in the PFSC, a biennial International Helping Families Change Conference (3 day), regional Triple P Master Classes (90 min), State-based Triple P Update Conferences (1 day), Podcast series for members of the public such as “Parenting in a Pandemic” (20 episodes) and “Families under Pressure” (12 episodes), and more recently leading the development of collaborative partnerships to host the inaugural International Congress on Evidence-based Parenting Support to promote policy and practice (www.i-ceps.pafra.net). The PFSC led the formation of the Parenting and Family Research Alliance (PAFRA) as a charity that brought together other evidence-based parenting programs in Australia to collectively advocate for increased research funding and policies to support the implementation of EBPS programs (e.g., Doyle et al., 2022).

### Implications for Other Programs

Building a credible evidence base for a multi-level system that involves a suite of programs rather than a single program takes time, resources and a sustained commitment of developers who are also researchers. Desirable independent evaluations inevitably delay the building of robust evidence due to factors such as programs needing to be sufficiently well developed with training mechanisms in place before programs can be shared. Other researchers have to be interested in the program, attract sufficient funding to conduct a sufficiently powered trial in a highly competitive grant environment that undervalued parenting intervention research (e.g., Havighurst et al., 2022), conduct the study and then publish the results in reputable peer-reviewed journals. This can take 5–10 years after the initial demonstration of the program’s effects. However, Fig. 1 shows it is feasible to build independent replication however only after a dissemination mechanism is established so that the program is accessible to others.

## The Present: Making a Population Approach Work at Scale

The past several decades has resulted in some key learnings about how to successfully implement the Triple P system at scale. This section summarizes these learnings.

### Importance of Involving Different Disciplines, Sectors, and Service Delivery Systems

To ensure a program has sufficient population reach EBPS should not be confined to a single discipline or service delivery sector. The involvement of multiple disciplines and service providers in delivering Triple P is both a strength and a limitation. Strengths include creating a common language and consistency of messaging and approach across different professionals supporting families and creating more destigmatized access points for parents for receiving help when and where it is needed (e.g., primary health care, early childhood education and care and schools, and community services). It is encouraging to note that the same standardized professional training program has been shown to produce similar positive training outcomes across different disciplines and sectors for satisfaction with training and practitioner self-efficacy (Sanders et al., 2022, 2023a; Sethi et al., 2014). An additional advantage of multidisciplinary training is greater awareness and appreciation of the complementary role of different disciplines and the potential to reduce interdisciplinary conflict “turf wars” over approaches. Some disadvantages include a lack of regulatory mechanisms and quality assurance e mechanisms to ensure ethical and professional standards are observed. Also, there are substantial differences in rates of reimbursement between disciplines for delivering the same or similar services to parents. In some LMIC’s parenting support is not a government priority and there is no dedicated workforce. Programs are unlikely to be scaled without the use of lay health workers who are likely to require co-facilitation and ongoing mentoring after initial training (Hodge et al., 2017).

### Having Clear Goals and Targets for Participation Across the Developmental Spectrum

The population approach aims to increase the number and proportion of parents who participate in an evidence-based parenting program and to decrease the number and proportion of parents who engage in coercive, dysfunctional, or unhelpful parenting practices. In order, to achieve such an outcome the participation rates of parents in parenting programs need to be reliably tracked. Capturing participation data is much more difficult than might be thought. First, there is lack of harmonization across sectors (health, education, welfare) in how program participation is defined and measured. Multi-sector integration of data sources from different agencies cannot occur without data-sharing agreements across jurisdictions. Without reliable measures of program participation and outcomes, it is difficult to know whether rates are changing (increasing, decreasing or staying the same) and whether a change in child and parent outcomes are being achieved. To overcome this problem, periodic population-based cross-sectional surveys of parenting and family well-being can be used to track parent reports of program participation over time (Morawska & Sanders, 2018. Such surveys need to target aspects of parenting that are linked to child outcomes and that can change because of intervention over time. This type of population data needs to be supplemented by service-based clinical outcome data using reliable, valid and change-sensitive assessment tools. ASRA (Automatic Scoring and Reporting Application) was developed to assist practitioners and agencies track client outcomes individually and at an aggregate agency level.

### Ensuring Multiple Levels of Intensity of intervention are Available

The rationale for having differing levels of intensity stems from the observation that parents have different needs and participate in programs at different points. For example, a parent who has already completed a multi-session parenting program such as Group Triple P and is looking for a brief or low intensity “booster” intervention with their next child, can be offered a seminar, discussion group or online option that consolidates and refreshes their prior learning and experience. Whereas a parent with no prior exposure positive parenting may benefit from a more intensive program particularly if they experience multiple problems in managing their child’s behavior.

### Encouraging Both Flexibility and Fidelity in Delivery

Poor or inadequate program delivery is a threat to achieving desired child outcomes. This occurs when practitioners fail to implement programs with sufficient fidelity (missed out essential procedures). However, the best client outcomes are likely to occur when both fidelity and flexibility are concurrently addressed (Mazzucchelli & Sanders, 2010). For example, videos, manuals, and workbooks typically include examples to illustrate parenting principles and strategies. These examples can be varied depending on participants characteristics (age group, type of problem, learning style) and by including functionally equivalent examples that are more locally relevant, salient, or culturally appropriate. An activity, exercise or homework task might take longer or less time depending on the skill level of participants. High quality implementation allows for the tailoring of both content and process fidelity to create a better “fit” of the program content to participants needs. However, it is not desirable to leave out recommended skills (e.g., quiet time or time out) because the practitioner does not like them or combine Triple P procedures with techniques from other programs with unknown efficacy.

### Having a Well-Trained and Supported Workforce

A workforce needs to be available to deliver enough programs to ensure the program reaches enough parents. Part of the challenge is that the delivery of parenting programs is typically not considered as an essential part of a professional role for many disciplines. Although different disciplines such as psychology, nursing, counselling, social work, and medicine can all be involved in delivering parenting programs for most professions being a parenting practitioner is not a primary clinical responsibility. This means the delivery of parenting programs is not prioritized and other core functions are typically given priority. For example, many school psychologists recognise the importance of parenting programs yet without a mandate from their school principal their time can often be dominated by case work and conducting of assessments for educational placements. Hence, organizational leadership is crucial to ensuring that trained providers achieve their implementation targets.

### Program Participation must be Inclusive

Parenting programs should be available to all parents/carers involved in raising children. This includes both parents (biological, adoptive and stepparents), grandparents, kinship carers, and foster carers. Although most participants in parenting research have been biological mothers, there is increasing evidence that fathers (Frank et al., 2015) and custodial and noncustodial grandparents also benefit from participation in parenting programs (Smith et al., 2018; Kirby and Sanders, 2014).

### Establishing Criteria for the Release of Programs

When should a new program be included in the Triple P system of evidence-based parenting support? Several different criteria need to be considered. These include the strength of the available evidence supporting the program. A well-executed RCT should normally be considered sufficient to justify the program to become part of Triple P and made available for dissemination. However, there are other important considerations, including the estimated costs of getting a program ready for dissemination (e.g., video production and printing costs, cost of translations and so on) and whether there is a market for the program. We have not always published programs we have developed and shown to be effective (e.g., a version of Group Triple P for parents of multiples) because of insufficient demand. A workforce needs to be identified and trained to deliver the program. The costs of making a program “dissemination ready” can be expensive and there must be a reasonable prospect of these upfront costs being recovered by the purveyor organization.

However, independent evaluation of a program is unlikely to occur unless the program can be disseminated. For third parties to evaluate the program they need to be able to access the official training, as well as program materials and resources to deliver the program. The release of the program heralds the possibility of further in dependent trials and evaluations which means the evidence-based is never static and continues to evolve. High quality service-based evaluations are particularly valuable as they show how well the program works in a normal service- delivery contexts and whether similar or better outcomes can be achieved. However, it can be a time consuming and expensive process required independent researchers to apply for and secure sufficient grant funding for an adequate statistically powered trial to be conducted.

### Adopting Culturally Informed Practices

Considerable recent attention has been focused on the issue of whether EBPS programs that were originally developed for parents in western HIC’s work or are culturally appropriate for other parents from non-English speaking countries or for ethnic minority, refugee, or First Nations parents. Increasing evidence shows that the core principles and strategies of positive parenting seem to work in a very diverse range of cultural contexts with relatively minor adaptations (Turner et al., 2020). Variants of Triple P has been trialled in culturally and linguistically diverse (CALD) populations in Australasia (Australia, New Zealand), United Kingdom (England, Scotland, Wales) and Jersey, North America (Canada, United States), Europe (Austria, Belgium, Denmark, France, Germany, Greece, Ireland, Luxembourg, Portugal, Sweden, Switzerland, the Netherlands), non-Western cultures in HIC’s including Asia (Hong Kong, Japan, Singapore) and South America (Argentina, Chile, Curacao) and LMIC’s in Europe (Romania), the Middle East (Iran, Turkey), Asia (China, India Indonesia, Malaysia, Vietnam), South America (Brazil, Columbia, Costa Rica, Mexico, Panama), Africa (Kenya, South Africa) and with Indigenous and First Nations families in Australia, Canada, New Zealand and the United States. Turner et al (2020) summarized program evaluations and outcomes for First Nations, non-Western/European and Culturally and Linguistically diverse (e.g. migrant and refugee) families and reported consistently high acceptability ratings and significant positive child and parent outcomes with varying levels of adaptation required.-from surface level (e.g. language translations, altered session length and structure, locally relevant teaching examples, culturally connected facilitators) to deep level cultural adaptations such as new or adjunct program resources that explicitly included cultural values. For example, teaching aids in the form of graphic representations linking the five principles of positive parenting with eight principles of Tikanga “ways of doing things”, experiential learning processes and tailored professional training (e.g., cultural supervision and mentoring by indigenous practitioners). In New Zealand considerable attention has been given to ensuring that the delivery of Triple P training uses culturally informed principles respectful of Māori indigenous culture. Another type of cultural adaptation involves how professional training is delivered to Indigenous practitioners. Triple P NZ employs a Māori cultural adviser to provide cultural supervision to contract trainers. It has also made adaptations to how training is conducted to be more attuned to working with Māori practitioners and whanau (families) in Aotearoa.

Another indicator of cultural acceptability relates to evaluations of professional training from different countries. A large-scale evaluation of Triple P Professional training courses showed very similar training outcomes across different countries and disciplines on measures of practitioner self-efficacy and program satisfaction (Sanders et al., 2022, 2023a). One factor that seems to contribute to achieving similar outcomes despite cultural differences is the adoption of a self-regulatory framework, that allows parents to choose their own goals. A parent’s goals can be informed by their own values and priorities. This means that parents can consider their own cultural understandings, beliefs, and traditions about parenting and children in formulating their goals. This approach is respectful of cultural differences and similarities providing parents with a range of tools that can be flexibly deployed in raising their children.

Several criteria can be considered in deciding whether to make surface or deeper level adaptations. These include whether existing surface-level adaptations are effective with the target population, the cultural acceptability to both parents and practitioners of programs, whether funding is available to support deep-level adaptations and the extent to which lack of adaptation would prevent program adoption or deployment.

### Making Local Adaptations

The statement “We are different here” captures a pervasive belief of many service providers that the parents they are working with are unique and differ in important ways from other parents, to require locally adapted versions of programs. This often translates into demands for solutions that are contextually relevant and directly related to the local population or setting. For example, delivery of Triple P in small rural towns can be very different to delivery in larger cities that have a more diverse and larger workforce. It is unlikely specialist programs for parents of children with developmental disabilities or parents of children who are overweight will be delivered by a specialist. However, adaptations can be expensive when new video footage is required and where published program resources need to be altered (tip sheets, workbooks). We routinely “Americanise” Triple P resources from Australian and British English to American English for spelling and to use local terms (nappies vs diapers; behaviour to behavior). It is not always possible to make such changes if the adapted program can only be used in a single location. Any major change or adaptation requires the consent of Triple P authors.

### Ensuring an Intervention is Ready for Scaling

To scale a multilevel system of intervention involves preparatory steps that require all the necessary program and training resources and materials to be available for dispatch and immediate use in sufficient quantities to meet the expected demands. Program and training materials should be professionally produced and safely stored in warehouse locations not prone to flooding. Resources (e.g., practitioner training manuals, parent workbooks, practitioner kits) must be packaged and dispatched in a timely manner to ensure they are available for use in training (often weeks in advance if being transported to an overseas country). Any customs requirements for sending materials across country borders need to be clarified and adhered to, to avoid border entry delays.

### Ensuring a Supportive Policy Environment

All parenting programs occur within a socio-political context that can be either favourable or unfavourable. A population-based parenting program requires a favourable policy environment for it to be sustained, so that dedicated funding for ongoing implementation and evaluation is recurrently budgeted for. Threats include lack of political advocacy for a program, changes of government or ministers and their priorities, that reduce funding available to support program implementation properly. An example of policy advocacy was the establishment of the Parenting and Family Research Alliance (pafra.org) in 2020 that specifically sought to change Government funding for research into parenting and the adoption of a population-based approach. One example of PAFRA’s work was to undertake a review of the level competitive grant funding focused on parenting through the Australian Research Council and the National Health and Medical Research Council in Australia over a decade. Of the 25,000 grants funded between 2010 and 2020 only 62 focused on parenting intervention accounting for only 0.25% of grants given. Other policy advocacy has included focused advocacy relating to the Medicare item numbers to enable parent only sessions to be conducted as part of children’s mental health plans. Another example is PAFRA advocacy pertaining to the National Disability Insurance Scheme (NDIS) for people with developmental disabilities. NDIS is funding model that made it more difficult for parent to access evidence-based group parenting programs (Mazzucchelli et al., 2023).

### Evolving a Sustainment Strategy

The successful implementation of a cost-effective system of intervention that produces policy-relevant outcomes is likely to be the most influential determinant of whether a program continues to be funded, however, success is no guarantee. The government’s priorities change because of economic circumstances and other priorities (e.g., pandemic, war, natural disaster). In many jurisdictions there seems to be little public accountability relating to parenting programs. For example, in Australia, it is unknown how much the federal and individual state governments spend on the implementation of evidence-based parenting programs and what outcomes are achieved. Bipartisan political support is highly desirable for the sustained implementation of evidence-based parenting support. Programs funded by government require continuous ongoing evaluation to promote quality improvement, public and unbiased reporting of outcomes achieved to promote greater public accountability.

### Addressing Risks and Threats to a Population Approach

#### Failing to Reach Enough Parents

A population approach is only likely to reduce the rates of targeted child problems if enough parents participate whose children are at risk. Although universal outreach to all parents helps to destigmatize program participation, other more targeted engagement strategies are needed as well. These strategies include peer-to-peer testimonial advocacy by similar parents who have participated in the program, advocacy by respected and well-known public figures, and delivery of parenting support in settings that parents already access for support in health, education, and social services.

#### Lower than Expected Rates of Program Delivery

Failure to achieve participation targets can also be related to lower-than-expected rates of program delivery by trained service providers. High staff turnover, lack of supervision or leadership support in the workplace, giving low priority to parenting program delivery compared to other clinical responsibilities and unforeseen disruptions due to the pandemic can all affect rates of program delivery. Practitioners will often struggle to reach parent participation targets if they only use a single mode of delivery. Running groups, seminars, and discussion groups and encouraging parents to complete online programs results in high rates of program participation than the delivery of programs to individual parents.

#### Unexpected Changes in Policies Affecting Service Delivery

Program delivery can be adversely affected by policy and administrative changes that decrease the access of parents to programs. For example, the introduction of the National Disabilities Insurance Scheme (NDIS) by the Australian Government provided care packages to individual clients. This inadvertently had the effects of reducing the capacity of community organizations delivering disability services such as Group Stepping Stones Triple P to offer and be reimbursed for delivering group parenting programs. Parents under stress because of children’s behavior cannot be expected to organize groups of other parents at the same time to participate so parenting programs can be delivered. Specific funding for Triple P Online in NZ by the Department of Health was introduced as a free service and achieved strong public take up during the pandemic but was subsequently withdrawn when the pandemic was seen to be over, even though children’s mental health problems stemming from the pandemic continued.

#### Not Being Able to Deliver Entire System

Population effects are more likely if a comprehensive range of programs are available. If only parts of the Triple P system are delivered particularly programs that relatively few parents participate in (e.g., Standard Triple P with individual clients in an agency) then the population reach of the intervention is likely to be too low to produce a detectable shift in prevalence rates at a population level even though individual families will benefit. In this situation additional resources will need to be found to either train staff to deliver a wider range of programs particularly group programs or make online programs available to parents.

#### Lack of Organizational Support

Organizational leaders in agencies need to support staff trained to deliver programs. If this does not occur, then lower rates of program delivery can be expected and therefore population reach will be insufficient. A change in leadership is a particularly high-risk transition time in implementing organizations. Active efforts to engage and gain the support of new organizational leaders is vital. Being able to produce convincing evidence of outcomes achieved with program delivery is likely to be important information in securing ongoing support.

#### High Staff Turnover and Retraining Costs

The costs of an intervention increase considerably if trained staff leave before they have become regular implementers of programs. A knock-on effect of failing to retain staff is that suitable replacement staff must be recruited and trained in one or more variants of Triple P. These additional training costs and time delays in accessing training need to be budgeted for based on expected retention rates of different types of staff in the organization.

#### Partnering and Working Alongside Other Programs

In most settings where Triple P is introduced for the first time as a population strategy, other parenting programs are often being delivered in the community. Triple P can be readily integrated into the service mix when it is seen as complementing, working with, and extending services available to families in a community. However, if the new program is seen as competing with and eventually replacing existing services, considerable local resistance can develop from well-established existing programs because it becomes a threat.

#### Implications for Other Programs

Systems-level parenting and family interventions that aim to “shift the needle” on population-level indices of child well-being or maltreatment need to synergistically bring together diverse disciplines, sectors, and service delivery systems that often operate in silos and compete for scarce resources. Deriving a shared vision regarding the goals and recognition of the need to adopt a population-based approach can be achieved when there are sufficient benefits for individual stakeholder groups including parents as consumers. Effective policy advocacy and strong local champions (other than program developers or purveyor organizations) can help combat bur not eliminate perceptions of bias and self-interest.

## Future Directions: Where to from here?

Many challenges must be addressed to ensure that EBPS reaches its full potential to promote the well-being of children and parents.

### Being Responsive and Adaptable

The global COVID-19 pandemic with its associated disruptions to “in-person” service delivery showed the crucial importance of evidence-based programs being able to rapidly pivot to new ways of engaging with families. Many in person parenting services had to rapidly and transition to telehealth or videoconferencing-based delivery of programs. The same occurred with training programs. For example, within a month of COVID beginning in February 2020 Triple P International transitioned the majority of its previously in person training programs to video conferencing-based delivery, without any adverse effects of training outcomes. Sanders et al., (2022, 2023a) compared training outcomes for in person training and zoom based training and reported no significant differences in practitioner satisfaction or practitioner self-efficacy following training. Although individual practitioners reported that due to poor internet access some individual families experienced difficulties, the overwhelming response of parents being able to access service from the convenience of their own homes was positive. For many organizations the convenience of flexible delivery using telehealth has become part of regular service delivery. However, the capacity of programs such as Triple P to be nimble and responsive requires a preparedness to change, financial resources and sufficient organizational capacity to make changes needed.

### Better Integration of Parenting Programs into Prevention and Treatment Services

Having a multilevel system of parenting support available for families with differing levels of intensity allows for better integration of parenting programs into prevention and treatment services. However, not uncommonly parents needing more intensive intervention find themselves falling between the gaps and ending up on long waiting lists. Efforts to reduce lag times between service sectors is not easily resolved, a task made more difficult by staff shortages in tertiary mental health services. As a response, online programs such as Triple P Online, Fear Less Triple P Online and Triple P for Baby Online can be used as a waitlist intervention for parents between initial referral and screening and the scheduling of intake interviews in the clinic.

### Collective Action to Address Policy-Related Obstacles

Policy-related obstacles such as lack of research funding for parenting research, administrative and logistical problems that decrease access of parents to parenting programs, and the lack of focus on parenting in mental health prevention and promotion activities can be addressed if there is greater collective action between researchers, programs developers and service providers. Effective policy advocacy needs to be independent of advocacy for specific programs and should focus on stakeholders coming together to advocate for the collective goal of increasing access to a range of evidence-based programs. Our experience with PAFRA has shown that respectful partnering and collaboration between different “competing” programs can achieve the dual goal of speaking with a united voice to government and supporting collective actions that benefit all programs (e.g., increased funding for parenting and family intervention research).

An increase in funding for research into parenting interventions is essential to ensure that parenting programs continue to evolve and produce solutions that address the contemporary concerns of parents. Without ongoing investment in research and development parenting programs can become outdated (e.g., materials such as video resources). Program innovation is necessary to address the evolving concerns of families (global warming, climate change, UN’s Sustainable Development Goals, cyberbullying) and how they wish to access programs. Greater investment in research and development should lead to a larger number of evidence-based parenting programs being developed, resulting in a greater range of options for practitioners and parents and more tailored programs addressing the needs of particularly groups (e.g., refugees, minorities, LGBTQI + parents).

### Developing Sustainable Models for the Scaling Parenting Programs

Within the wider landscape of dissemination of evidence-based parenting programs, different purveyor models have emerged to scale interventions. All dissemination efforts must be based on sound business principles so that the costs of program dissemination can be covered and that sufficient funds are generated to enable ongoing investment in future program development. Dissemination efforts undertaken by developers or purveyor organizations on behalf of developers are more likely to be sustained when based on sound financial principles. This applies to any developer whether they work in the University sector, independent research organizations, charities/not-for-profit, and for-profit commercial organizations This is particularly the case when multi-level systems of intervention are developed that have many moving parts and that require ongoing research and development investments to maintain. Triple P is an example of a dissemination model that has been sustained for over 25 years. The University of Queensland employs the staff who developed Triple P and own the intellectual property vested in Triple P. After our initial efforts to disseminate through the Parenting and Family Support Centre, we collaborated with UniQuest (UQ’s main technology transfer company) who commercialized the dissemination of Triple P by providing an exclusive global license to a startup purveyor company Triple P International to scale the program on behalf of the University. Having an industry partner dedicated to disseminating Triple P was a game changer. It eventually produced a revenue stream through royalties that has helped to sustain and evolve the program over time. Royalties arising from TPI’s training, and implementation support and sale of program resources are split according to the Universities Intellectual Property Policy between UniQuest, the Faculty of Health and Behavioural Sciences, the Parenting and Family Support Centre in the School of Psychology, and approximately 13 contributing authors. This arrangement has enabled the program to be disseminated globally. This financial model incentivises all parties and has enabled the program to develop new variants. Many evidence-based parenting programs do not get disseminated effectively because of an inadequate, non-sustainable financial model.

### Improving Pre-service Training of Professionals

Most disciplines involved in delivering parenting programs including psychology, social work, nursing, medicine, psychiatry, counseling and education professionals do not provide competency-based professional training to students in delivering evidence-based parenting support. Most professional training in the parenting field occurs through “on-the job” in-service training. The lack of preservice training means that many students have very limited theoretical or practical knowledge or clinical skills before entering the workforce and report low self-efficacy about how to consult effectively with parents. The provision of foundational training in University courses in at least one evidence-based program would be a valuable addition to any training of professionals to work with children and families. At the University of Queensland, Clinical Psychology and Counselling students receive accreditation-based training in at least two different Triple P programs as part of their University course (usually Standard or Group Triple P). This training helps prepare students for working with clients in internships.

### Defining Child and Parent/Carer Capabilities Associated with Patterns of Sustainable Living

Little attention has been given to the potential role of parenting interventions in promoting child, parent and family capabilities related to sustainable development. Sanders et al., (2022, 2023a) argued that the attainment of the United Nations Sustainable Development Goals (SDGs) requires the scaling of parenting interventions. Many individual SDGs are more likely to be achieved if children and their parents/carer acquire complementary and, in some cases, reciprocal capabilities that support patterns of sustainable family living. WHO (2022) recently identified eight of the 16 SDGs linked to parenting including Target 16.2 “End abuse, exploitation, trafficking, and all forms of violence against children and torture of children” and target 4.2 “Provide access to quality early childhood develop and care”.

### Improving Knowledge Transfer and Science Communication

Evidence-based parenting support would have greater impact and public policy support if the discoveries and key findings from parenting and family intervention research were disseminated more effectively to a wider audience. Traditional methods of knowledge transfer through publication in scholarly journals and professional conferences have limited impact. Additional methods of knowledge transfer include the preparation of policy briefs, greater use of social media tools (twitter), the development of themed vodcasts and podcasts dedicated to parenting topics and issues, greater use of global digital congresses and conferences to enable wider participation, particularly by stakeholders from low- and middle-income countries. A recent example of such an approach was the inaugural International Congress on Evidence-based Parenting Support held in June 2023. This 3-day virtual congress included a range of communication strategies designed to promote post congress policy and social action in policy, research and practice following the congress. It included creating small “Action Circles” to work collectively on solving problems, special issues of journals, podcasts, and access points for practitioners and agencies to connect with evidence-based programs and researchers (see i-ceps.pafra.org).

### Keeping Track of Global Change Through a World Parenting and Family Well Being Survey

A global Parenting and Family Wellbeing Survey with a representative sample of families of children in different age groups is needed to enable progress in addressing parenting and family issues to be monitored and tracked at a country level over time. These essential epidemiological data could then be linked to other data sets (health, education, welfare) pertaining to the wellbeing and children and families. The lack of an agreed upon reliable, valid and change sensitive epidemiological survey tool precludes any country comparisons being made. Although individual state jurisdictions have conducted such surveys Victorian Parenting Survey (Wade et al., 2018) and the Every Parent Survey (Sanders et al., 2005) the resulting data could be put to greater use in influencing national policies affecting children and families.

### Opportunities Using New Technologies to Share Knowledge

The pace of growth of AI (Artificial Intelligence) technologies has increased exponentially over the past few years. The next generation of professional training programs is likely to rely increasingly on technology including AI to reduce the costs of training and to provide augmented training experiences for a wider range of professionals. Although AI is unlikely to replace all in person professional training those aspects of training that involve sharing information about theory, principles and evidence lend themselves to the use of AI. Training elements that involve skill development through coaching involving practice and feedback are likely to be more difficult particularly when the skills being taught to practitioners are complex, multi-phased and highly dependent on sequencing of a parent’s response to prior prompts and feedback provided by practitioners. Notwithstanding the challenges our team is involved in ongoing research to examine the use of technologies to improve professional training including virtual reality. EBPS interventions are likely to continue to evolve with a sustained commitment to ongoing research and development.

### Changing the Language of Parenting Support

Historically, the term parent training served the field well and differentiated behavioral approaches to parenting support from other theoretical approaches (based on psychodynamic or attachment theory) and more general parent education (e.g., playgroups). However, over time, the term has become more of a liability, and it should be replaced by the more generic term evidence-based parenting support for the following reasons. First, many parents with concerns about their children’s behaviour who seek professional support do not see themselves as requiring ‘training’ or ‘therapy’. Second, the traditional intensive group and individual programs used with parents of children with severe conduct problems capture only part of the evidence base used to promote better outcomes for families. In particular, the growth of low-intensity parenting seminars, discussion groups and online programs require more inclusive language based on the principle of proportionate universalism. Its meaning in the context of parenting intervention is that everyone is likely to need some support, with more intensive interventions limited to families with greatest need or who are non-responders to less intensive interventions.

### Understanding Mechanisms of Change and Non-response to Intervention

The current prevention and treatment technologies that use EBPS are not a panacea and there are always non-responders to any intervention. More research is needed to identify early antecedents and indicators of premature dropout from an intervention or non-response among completers of the intervention. Also, social learning theory and contingency management principles have featured strongly in the theoretical base of parenting programs with parents and as a central explanation for improvement in child behaviour (parents become more positive and contingent and less coercive and unpredictable). However, there are other possible explanations for change in both parenting and child outcomes that should be considered in any analysis of mediators of change. These include cognitive factors such as changes in parental expectations and attributions, changes in available social support (less couple conflict, better teamwork), changes in parent adjustment (less stress, depression, anxiety) and the therapeutic alliance (relationship with practitioner). Research is needed that tracks change over time on multiple variables during intervention to identify the timing, sequencing and patterning of change on different putative mediators. Some variables may change early, others change later, and some not at all over the course of an intervention. Apart from mechanisms explaining change in parenting, the factors that influence whether trained and accredited practitioners implement Triple P requires further investigation.

### Using Diverse Research Methods

The field of parenting intervention has benefitted from a wide range of methodologies, and this should continue. For example, research in Triple P has ranged from N = 1 experiments to randomized clinical trials, place-based population trials, quasi-experimental evaluations, service-based evaluations, and qualitative studies (focus groups, interviews) examining cultural acceptability, economic analyses, and meta-analyses. More population-level evaluation studies with longer periods of follow-up are needed to strengthen findings regarding the population-level effects of the Triple P system on child maltreatment and rates of social, emotional, and behavioral problems. However, when evidence-based parenting programs are widely disseminated and become an integral part of “care as usual”, different types of control conditions are needed and designs such as non-inferiority RCTs are more likely to be used (Prinz et al., 2022).

### In Search of More Effective Parenting Strategies to Improve Child Outcomes

Many techniques used in positive parenting programs have been available for decades (e.g., providing clear instructions, praise, and timeout). However, few studies have investigated how to further improve the effectiveness of specific techniques, or combinations of techniques used (e.g., quiet time, timeout, descriptive praise, incidental teaching, reward charts). Salmon et al (2014) showed that adding emotional literacy training for parents did not improve the effects of Group Triple P with parents of disruptive preschool-aged children. Procedures that enhance positive home-school communication is another area for innovation. Many educators are concerned that too many children are inadequately prepared to begin formal schooling. Many children at school entry have delays in language, pre-literacy skills, self-care skills (independent toileting), poor emotion regulation and significant social and behaviour problems. All these difficulties can be influenced by parenting. Also, brief parenting and teacher-training programs focused on improving home-school communication seem particularly valuable.

### The Need for Branded Interventions

Criticisms of commercially disseminated programs such as Triple P, Incredible Years, MST and PCIT because they are ‘branded therapies’ seems misplaced. The approach advocated by some involves the identification of common elements of effective intervention and disseminating them as non-branded or non-commercialized programs. However, all programs eventually acquire an identity and “brand name” so they can be effectively promoted to parents and professionals. Furthermore, a sustainable model of dissemination requires an economically viable business model where revenue from training and sales of program resources and materials and implementation consultation helps recover the real costs of developing and maintaining an evidence-based program (e.g., revisions and updates of professional and parent resources, written materials, videos, online programs). Without competition there are few incentives for program developers to reduce or contain costs and training and dissemination activities become wholly reliant on subsidies from the government or foundations that are usually not sustainable.

### Using the Internet to Improve Population Reach

The internet has markedly increased the capacity of people to share useful information, rapidly, conveniently, and at no or low cost. The internet via social media (e.g., Facebook, Twitter, blogs, online forums, and parenting networks) has increasingly been used to encourage participation in parenting programs (Baker et al., 2017). However, parents are bombarded with many messages about parenting requesting their time or money. Greater attention needs to be given to identifying the best ways to create messaging that gain a parents’ attention and motivates them to participate in programs proven to work. Peer-to-peer advocacy can be a powerful inducement for parents to register for a parenting program. However, is it better to promote a program as something that will alleviate a child’s problem behavior or promote the child’s well-being and success in life? At present it is unknown what kind of parenting messages work best to encourage parents to participate in large numbers.

### Using Evidence to Influence Policy

Much greater focus is needed to ensure that available evidence is considered by policy makers when making funding decisions. Few professional training or higher degree training programs provide specific training in policy analysis or how to use evidence to influence policy decisions and policy makers. Skills such as how to effectively engage with and communicate scientific findings to different audiences (e.g., policymakers, mass media, and consumers) are rarely taught in graduate training programs.

### Parenting Programs and Adult Mental Services

Many adults with serious mental health problems are also parents, and their children can be at risk of mental health problems themselves because of disruptions to family life and parenting (neglect) caused by parental mental illness (Calam et al., 2017). Parents with mental health problems can benefit from participation in positive parenting programs. For example, Jones et al. (2017) used the 8-module Triple P Online along with an 8-module online CBT program for parents with bipolar disorder and were able to demonstrate fewer mental health problems in children. Parenting interventions are trans-diagnostic interventions that can be applied to a wide range of problems, diagnoses, and conditions. As programs such as Triple P have five core principles that are relevant to all children and 17 different strategies that can be customised to the specific concerns of parents, there is great potential for these interventions to be flexibly adapted to address the unique parenting context the parent is experiencing. To avoid the unnecessary proliferation of multiple program variants, practitioners need to be trained to flexibly apply principles and procedures to the unique circumstances of parents irrespective of diagnosis based on a case formulation. Different variants are justified if a different skill set is needed by the parent (e.g., parenting teenagers or children with a disability) and there is additional content that a practitioner needs to understand (e.g., Lifestyle Triple P with parents of obese children). Although social learning theory and the principles of behaviour change have been largely derived from the discipline of psychology, for parenting programs to make a population-level impact on problems such as child maltreatment and mental health, multiple disciplines that have a mandate in their work role to provide parenting support need to be used to reach parents. In low resource settings where there are simply very few highly trained mental health specialists such as psychologists, social workers or nurses, appropriately trained and supervised non-specialist volunteers may be needed (Ward et al., 2015). This approach has been successfully used in some low and middle-income countries such as India to deliver CBT interventions for depression and alcohol problems (Chowdhary et al., 2016). However, use of volunteers does not eliminate the need for sustainable system of workforce training and supervision.

### Responding to Criticisms

Although EBPS has flourished and has been widely adopted, it has also received some criticism. One of the most voiced criticisms is that discipline methods such as timeout damage the parent–child attachment relationship and that their use is contraindicated in children with histories of trauma. There is no evidence to support this contention when timeout is used appropriately, and there are literally hundreds of well-controlled RCTs evaluating parenting programs that have used timeout showing positive effects on children’s behaviour. The responses to these criticism from supporters of timeout have included writing articles that challenge the criticism and clarify the conditions for effective usage, conducting literature reviews of available literature, and challenging conclusions in professional forums (Dadds & Tully, 2019; Morawska & Sanders, 2011). However, an alternative response could be to search for ways of using timeout that reduces the intensity and duration of distress and protest the child engages in (e.g., using behavioural rehearsal to prepare the child in advance, reminders of rules that apply in specific situations, avoidance of threats, warnings, or voice escalation, use of shorter periods of timeout, increasing the contrast between time in and timeout by increasing positives for prosocial behaviour).

Other criticisms have been methodological, such as foundational trials being under powered, lack of independent evaluations by non-developers, and failure to disclose conflicts of interest (COI) in publications. Some criticisms have been valid and have resulted in a change of practice particularly with respect to routine COI disclosures (see Sanders, 2015; Sanders et al., 2019, 2020b). For example, lack of transparency with respect to financial benefits developers might receive from dissemination of a program has been a problem. However, the situation appears to be changing with a much higher level of disclosure of COI occurring in more recent years. Disclosure of COI, however, does not obviate the need for conflict-of-interest management practices to be in place and for best practice guidelines to be developed to eliminate or reduce research bias (Sanders et al., 2019).

### Trauma-Informed Intervention

There have been increasing calls for services to identify themselves as ‘trauma-informed’ and to offer so-called ‘trauma-sensitive’ parenting interventions. This movement stems from the work of Felitti et al. (2009) in the United States, who identified that an individual’s family of origin exposure to adversity in childhood increases lifetime risk of a range of serious mental and physical health problems. However, the specific branding of services, agencies, programs and professionals as trauma-informed to differentiate them from other services is misleading and unnecessary. It is like branding a program as gender sensitive and culturally informed. All professionals need to be aware that problems arise in diverse contexts and that a thorough history and case formulation should seek to identify past and current life circumstances and events, including trauma that have shaped the nature and severity of a parent or child’s problem. Recognising that a child or parent may have been exposed to prior trauma does not automatically lead to a list of separate clinical actions that are either trauma informed or not trauma informed. Rather, everyone should be striving to respond appropriately to the assessed needs of clients in context including whether the client provides a mandate to address the trauma.

### Implications for Other Programs

Evidence-based programs must continually evolve to remain relevant to the contemporary and changing needs of children, parents and communities. The COVID-19 pandemic taught program developers that flexible delivery and that telehealth and online programs could be effectively used when both practitioners and parents were in lockdown. However. The concerns about existential threats due to climate change (e.g., natural disasters), global threats to democracy because of war, internal displacement, and violence have had serious impacts on children and their families. New interventions are needed, and older ones need to be adapted to address such concerns.

## Conclusion

Of the many potential actions that could be taken to improve the life course outcomes of children, none are more important than improving the knowledge, skills and confidence of parents in the task of raising their children. When all parents are encouraged to embrace this collective goal, everyone is likely to benefit-children, parents, schools and the wider society. Evidence-based parenting support needs to become a policy priority to reduce the prevalence rates of social, emotional, and behavioral problems and rates of child maltreatment. Receiving evidence-based parenting support needs to become socially normative, stigma free and viewed by the wider community as something that is healthy, desirable, and associated with benefits for all (children, young people, parents and carers themselves and the wider community). Each country and local jurisdiction should set and measure the attainment of specific goals and targets relating to parent participation and efforts made to reduce barriers to access and to encourage participation of all relevant parents and carers.

Over the past four decades, the population approach to parenting support has gradually evolved from targeted individual and group programs developed in the 1960s and 1970s for children with conduct problems to a more sophisticated multi-level system of parenting support capable of addressing a wide range of parenting concerns. Despite growing evidence that parenting programs work, are cost-effective, can be scaled, and can be applied to a diverse range of social, emotional, and behavioral problems there is still significant underinvestment in most countries to make parenting programs widely available to families. Quality parenting programs should become the centrepiece of programs to prevent social, emotional, and behavioral problems and prevent child maltreatment and be incorporated as appropriate into child-focused, family centered treatments. Much greater investment in ongoing research and development is needed to ensure that programs continue to evolve, address the changing contemporary concerns of parents, and consider the developmental changes associated with the life course journey that individual parents experience for better or worse**.** This journey starts when parents first transition to parenthood and continues throughout their life.

Finally, implications for the wider field of evidence-based child and family interventions stem from our experience with Triple P. Our program benefits from having a sustained organizational commitment to evolve and sustain the program over time as a comprehensive multi-level system of intervention with programs that address a full spectrum of intervention from early prevention infancy through to more treatments for complex, vulnerable families. Program developers and leaders of Centers involved in program development must address the never-ending challenge of securing sufficient funding to support necessary infrastructure so that long-term strategic planning is possible (including legacy planning when original developers retire or leave). Programs such as Triple P should not be reliant on the continued involvement of original developers. The next generation of capable early-career and mid-career researchers who are also program developers can then step up to continue the work, improve programs and build on the foundations established by their predecessors and mentors.
